# Quantification of Total Free Radicals in *Drosophila* Using a Fluorescence-Based Biochemical Assay

**DOI:** 10.21769/BioProtoc.5238

**Published:** 2025-03-05

**Authors:** Shahira Helal Arzoo, Rubaia Tasmin, Surya Jyoti Banerjee

**Affiliations:** Department of Biological Sciences, Texas Tech University, Lubbock, TX, USA

**Keywords:** Free radicals, Oxidative stress, *Drosophila melanogaster*, Fluorescence assay, High throughput, Dichlorofluorescein

## Abstract

Free radicals, including reactive oxygen species (ROS) and reactive nitrogen species (RNS), induce oxidative stress. This stress plays crucial roles in cellular signaling, stress response, and disease progression, making the quantification of free radicals essential for understanding oxidative stress mechanisms. Here, we present a high-throughput fluorescence-based protocol for measuring the presence of total free radicals, including ROS and RNS, in the whole adult *Drosophila melanogaster* (fruit fly). The protocol involves homogenizing whole adult flies in PBS and treating only the supernatant of the lysate with dichlorodihydrofluorescein-DiOxyQ (DCFH-DiOxyQ), which then converts into a fluorescent molecule, dichlorofluorescein (DCF), upon reacting with free radicals. The level of fluorescence is directly proportional to the amount of free radicals present in the sample. This protocol offers simplicity, scalability, and adaptability, making it ideal for studying oxidative stress in the model organism *Drosophila* and its different tissues under different dietary regimes, environmental stresses, genetic mutations, or pharmacological treatments. It is to be noted that the protocol uses a kit from Abcam, which has been used to measure free radicals in mice, rats, human blood, and cell lines. It can also be applied to biofluids, culture supernatants, and cell lysates, making it suitable for a wide range of sample types beyond whole organisms or tissues. However, due to our research focus and expertise, here we describe a detailed protocol to measure free radicals responsible for inducing oxidative stress only in fruit flies.

Key features

• Quantifies total free radicals including ROS and RNS levels in adult *Drosophila melanogaster* using a fluorescence-based approach for oxidative stress studies.

• Suitable for high-throughput analysis with a 96-well black plate format, simultaneously enabling efficient handling of multiple samples and standards.

• Adaptable to different experimental conditions, including diverse ROS-inducing treatments and mutations in *Drosophila*.

• Offers detailed instructions for reagent preparation, sample homogenization, fluorescence measurement, normalization, and statistical analysis of data to ensure reproducibility and accuracy across research settings.

Graphical overview

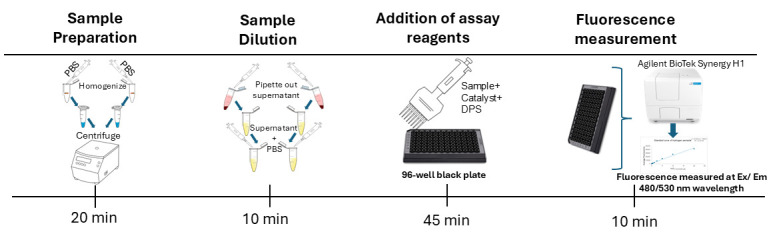

**Schematic workflow of the assay.** Whole adult fruit flies are homogenized in PBS buffer and centrifuged. The clear supernatant is carefully transferred into new tubes for further treatment with different reagents, loaded into a clear-bottom black 96-well plate, and treated with another set of reagents. The plate is then incubated, and fluorescence is measured using the Agilent BioTek Synergy H1 plate reader.

## Background

Free radicals, including reactive oxygen species (ROS) and reactive nitrogen species (RNS), are crucial players in cellular homeostasis [1]. Their imbalance contributes to numerous pathological conditions [2], aging [3], cancer [4], and neurodegenerative diseases [4]. ROS and RNS are byproducts of normal metabolic processes [5], primarily from mitochondrial respiration [6]. However, excessive ROS levels or diminished antioxidant defenses cause oxidative stress, which damages proteins, lipids, and nucleic acids [7]. This disruption is observed in several diseases, including Alzheimer's disease [8], Parkinson's disease [9], and various cancers [10]. Research in model organisms like *Drosophila melanogaster* has greatly advanced our understanding of ROS-related pathophysiology [11].

Several existing methodologies are employed to measure ROS, including chemiluminescence [12], electron spin resonance spectroscopy [13], and flow cytometry [14]. These techniques are highly sensitive but very complex and often require advanced instrumentation, expensive reagents, and specific expertise. Furthermore, they may not be readily adaptable to all experimental designs or accessible to all laboratories. Thus, the need for a simple, scalable, and cost-effective alternative method remains a critical challenge in oxidative stress research.

**Figure 1.**
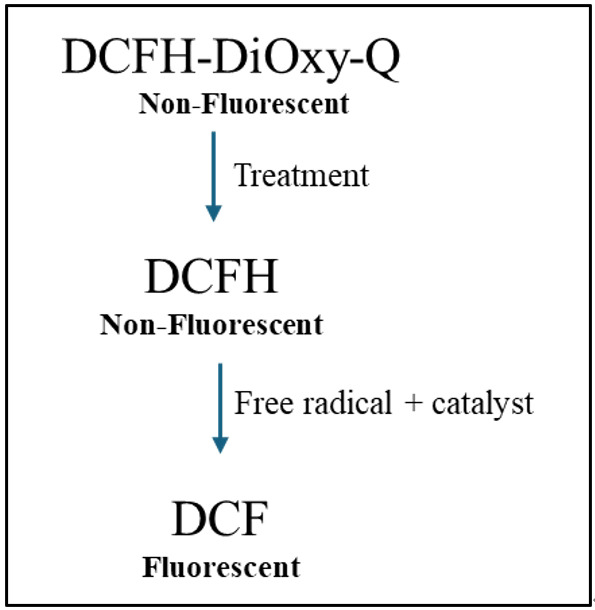
Mechanism of in vitro reactive oxygen species (ROS)/reactive nitrogen species (RNS) assay. Dichlorodihydrofluorescein-DiOxyQ (DCFH-DiOxyQ), a non-fluorescent quenched dye, is treated first with a priming solution to form DCFH-DiOxy. Treatment with a stabilization solution converts it into DCFH, which remains non-fluorescent. In the presence of ROS/RNS and a catalyst (such as an esterase), DCFH is oxidized to fluorescent dichlorofluorescein (DCF), allowing ROS/RNS detection [19].

For our assay, we used three biological replicates, each with two technical replicates following the common practice in biochemical assays using fruit flies [15–17]. Our protocol employs a fluorescence-based approach for quantifying free radicals (ROS and RNS) in *Drosophila*. This method involves preparing samples by homogenizing whole adult flies, preparing hydrogen peroxide standards, and treating the samples and standards with a non-fluorescent dye, dichlorodihydrofluorescein diacetate (DCFH-DA), which generates the fluorescent product DCF upon reacting with free radicals such as ROS and RNS in the presence of a catalyst (ab238535) [18,19]. Fluorescence is measured using excitation/emission wavelengths of 480/530 nm using a plate reader (Figure 1). The fluorescence of each sample is used to calculate the concentration of free radicals in the sample by using the formula generated in the standard curve analysis. We use the Bradford assay to measure the protein concentration of each sample and normalize the data by dividing the free radical concentration by the protein concentration of the corresponding sample. This is a common practice for biochemical assays [15–17] to calculate the relative amount of free radicals per unit of protein. Normalization of data helps to avoid false differences in fluorescence levels between two samples due to different dilutions as a result of human and technical errors.

A significant advantage of this protocol is its accessibility and scalability, requiring only standard laboratory equipment and reagents. It allows researchers to investigate oxidative stress in various biological contexts, including dietary intervention, the impact of genetic mutations, environmental stressors, and therapeutic interventions in *Drosophila*. Although this protocol does not quantify specific types of free radicals, it provides a reliable measure of overall ROS and RNS levels in flies, which is often sufficient for many experimental objectives. With its simplicity and versatility, this protocol represents a significant step forward in the field of oxidative stress research in fruit flies.

## Materials and reagents

**Biological materials**

1. *Drosophila melanogaster* (collected from Bloomington Drosophila stock center)

**Reagents**

1. DCF ROS/RNS assay kit (Abcam, catalog number: ab238535); contains enough reagents to perform 96 assays (Table 1)

**Table 1.** Storage conditions, concentrations, and volume of individual components from the kit

Item	Stock concentration	Volume	Storage conditions
Priming reagent	1×	250 μL	4 °C
Stabilization solution	10×	1.5 mL	4 °C
Catalyst	250×	20 μL	4 °C
DCF-DiOxyQ	40×	50 μL	-20 °C
Hydrogen peroxide (H_2_O_2_)	4,400×	100 μL	4 °C

2. Bovine serum albumin (BSA) (Sigma-Aldrich, catalog number: A7906-50G)

3. Bradford reagent (Thermo Scientific, catalog number: 23238)

4. Sodium chloride (NaCl) (Sigma-Aldrich, catalog number: S7653)

5. Potassium chloride (KCl) (Sigma-Aldrich, catalog number: P9333)

6. Disodium phosphate (Na_2_HPO_4_) (Apex, catalog number: 20-147)

7. Potassium phosphate monobasic (KH_2_PO_4_) (Sigma-Aldrich, catalog number: P5655)

**Solutions**

1. 1× stabilization solution (see Recipes)

2. 1× catalyst (see Recipes)

3. 1× DPS (see Recipes)

4. Hydrogen peroxide standard (see Recipes)

5. BSA standard (see Recipes)

6. 1× phosphate buffer saline (see Recipes)

**Recipes**

*Note: Recipes below are for 50 reactions (one reaction per well of a 96-well plate). Prepare a little extra volume than the exact volume required to make up for any pipetting error.*

**1. 1× stabilization solution (5.5 mL)**

Mix 550 μL of 10× stabilization solution (from the assay kit) with 4,950 μL of deionized water to make 1× solution. Vortex and store at 4 °C until use.

**2. 1× catalyst (2.75 mL)**

Mix 11 μL of 250× catalyst (from the assay kit) with 2,739 μL of 1× PBS (Recipe 6) to prepare 1× catalyst solution. Vortex and use immediately.

**3. 1× DPS (2.75 mL)**

Mix 27.5 μL of DCF-DiOxyQ with 110 μL of priming reagent. Vortex and incubate at room temperature for 30 min. Then, add this mixture to 5362.5 μL of 1× stabilization solution (Recipe 1). Cover the tube in aluminum foil. Store on ice for immediate use. Any excess can be stored at -20 °C for up to a week for future assays.

**4. Hydrogen peroxide standards**

**Critical:** Freshly prepared sets of standards are made in tubes wrapped with aluminum foil. Prepare a 2 mM H_2_O_2_ stock solution by mixing 1 μL of 4,400× H_2_O_2_ with 4.4 mL of deionized water. This stock solution is used to prepare standards. See Table 2 for H_2_O_2_ standard preparations with 1× PBS (Recipe 6).

**Table 2.** Hydrogen peroxide standards

Standard #	2 mM H_2_O_2_ Standard (μL)	1× PBS (μL)	H_2_O_2_ conc. (μM)
1	10	990	20
2	500 μL of standard 1	500	10
3	500 μL of standard 2	500	5
4	500 μL of standard 3	500	2.5
5	500 μL of standard 4	500	1.25
6	500 μL of standard 5	500	0.625
7	500 μL of standard 6	500	0.313
8	0	1,000	0 (blank)

*Note: In this protocol, the standard curve for hydrogen peroxide has been adjusted from the original range of 0.039 μM to 0.313 μM, as specified in the protocol for the ab238535 kit (Abcam). This modification is based on the observation that the lower concentration range (0.039 μM) is not necessary for the sensitivity and detection limits of the assay under the conditions used in this study. In our sample, the free radical concentration equivalent to H_2_O_2_ concentration in the standard curve fell between 10 and 20 μM. However, researchers can use lower concentrations of H_2_O_2_ in the standard curve as suggested by the Abcam protocol if they use a lower number of flies or a higher dilution of fly samples.*

**5. BSA standards**

Prepare 2 mg/mL BSA stock solution by combining 40 μL of BSA stock (50 mg/mL) with 960 μL of 1× PBS (Recipe 6), yielding a total volume of 1,000 μL. Then, prepare a series of standard dilutions from the 2 mg/mL BSA stock solution with varying concentrations in cooled 1× PBS as shown in Table 3.

**Table 3.** BSA standards

BSA	Concentration (mg/mL)	Volume from previous standard (μL)	1× PBS (μL)	Remaining total volume (μL)
Blank	0	0	25	25
Standard 1	0.125	25 of 0.25 mg/mL	25	50
Standard 2	0.25	25 of 0.5 mg/mL	25	25
Standard 3	0.50	25 of 1 mg/mL	25	25
Standard 4	1.00	25 of 2 mg/mL	25	25
Standard 5	2.00	50	0	25

**6. 1× phosphate buffer saline (PBS)**

a. Begin by making a 10× PBS solution, placing 250 mL of distilled water in a 500 mL container.

b. Add the following reagents to the distilled water:

• 80.06 g of NaCl (1.37 M)

• 2.01 g of KCl (27 mM)

• 26.8 g of Na_2_HPO_4_ (100 mM)

• 2.45 g of KH_2_PO_4_ (18 mM)

c. Adjust the pH of the solution to 7.4 using a pH meter.

d. Add distilled water to bring the total volume up to 500 mL.

e. Aliquot prepared 10× PBS into five bottles, each containing approximately 100 mL of PBS. Sterilize by autoclaving for 30 min at 121 °C.

f. Dilute the 10× PBS stock solution 1:10 with distilled water. For example, mix 50 mL of 10× PBS with 450 mL of distilled water to make 500 mL of 1× PBS.

**Laboratory supplies**

*Note: Similar lab supplies from other companies will work as well.*

1. 96-well transparent plate (Genesee Scientific, catalog number: 25-109)

2. 96-well black plate (Thermo Scientific, catalog number: 165305)

3. PolyPestle (Genesee Scientific, catalog number: 25-280)

4. Microcentrifuge tube (clear polypropylene 1.7 mL tube) (Olympus, catalog number: 24-282)

5. Single (Sartorius brand, 1–10, 10–100, 20–200, 100–1,000 μL) pipette and multi-channel pipettes (Poseidon 8 Channel P300 Pipettor, 20–300 μL)

6. Sterile tips (Olympus brand, 10, 100, 200, and 1,000 μL tips)

7. Pasteur pipette (VWR, catalog number: 14672-200)

## Equipment

1. Agilent BioTek Synergy H1; filter set: excitation at 480 nm and emission at 530 nm

*Note: Any other plate reader that can measure the above fluorescence wavelengths can be used.*

2. Microcentrifuge (Hermle, catalog number: Z216-MK)

3. Vortex (Benchmark, catalog number: BV1000)

4. Mini centrifuge (Genesee Scientific, catalog number: 27-523)

5. Autoclave (Priorclave, catalog number: 150-320L)

6. -80 °C freezer (Eppendorf, catalog number: EPP: F740540015)

7. -20 °C freezer (VWR, catalog number: 76580-128)

8. pH meter (Thermo Scientific, model: Orion Star A211)

## Software and datasets

1. GraphPad Prism (https://graphpad-prism.software.informer.com/7.0/)

2. Gen5 3.14 ( https://www.agilent.com/en/support/biotek-software-releases)

3. Microsoft Excel

## Procedure

**A. Preparation of fly homogenate**

1. Rinse 25 anesthetized flies per biological replicate in 500 μL of cold 1× PBS in a 9-well glass plate to eliminate any food residues clinging to their exterior.

2. Transfer the flies to a 1.5 mL microcentrifuge tube.

3. Quickly spin the tubes for 5 s in a tabletop mini centrifuge.

4. Carefully remove all traces of liquid using a Pasteur pipette.

5. Make lysate of the whole adult flies by grinding them in 500 μL of ice-cold 1× PBS in a 1.7 mL microcentrifuge tube using a PolyPestle. Homogenize the lysate by gently pipetting 5–10 times (a motor can be used to facilitate homogenization)

6. Centrifuge the homogenate in the tube at 10,000× *g* for 5 min at 4 °C to precipitate the insoluble fly body parts.

7. Carefully collect the supernatant (~350 μL) in a new microcentrifuge tube by pipetting. The supernatant will be used to analyze ROS and RNS levels.

8. Dilute 50 μL of the supernatant by adding 150 μL of ice-cold 1× PBS (4 times dilution of the supernatant).

9. Mix the diluted supernatant thoroughly before further analysis or store it at -80 °C for one week.

**Caution:**

1. Always keep samples on ice during the procedure to prevent enzymatic activity and degradation of biomolecules.

2. During liquid removal, avoid aspirating the pellet, which can contain insoluble debris.

**Pause point:** If necessary, collected supernatant can be stored at -80 °C for up to one week before proceeding with the assay.

**B. Quantifying total free radicals**

1. Add 2× 50 μL (for two technical replicates) of sample or hydrogen peroxide standard solution into the wells of a clear bottom black 96-well plate by pipetting. See Table 4 for an example of a plate layout.

**Table 4.** Plate layout example with hydrogen peroxide standard solutions and samples, in a clear-bottom 96-well black plate for ROS and RNS assay

	1	2	3	4	5	6	7	8	9	10	11	12
A	ST.1	ST.1	CF1	CF1	CM1	CM1						
B	ST.2	ST.2	CF2	CF2	CM2	CM2						
C	ST.3	ST.3	CF3	CF3	CM3	CM3						
D	ST.4	ST.4	MF1	MF1	MM1	NM1						
E	ST.5	ST.5	MF2	MF2	MM2	MM2						
F	ST.6	ST.6	MF3	MF3	MM3	MM3						
G	ST.7	ST.7										
H	BLK	BLK										

ST. = standard; CF = control female; MF = mutant female; CM = control male; MM = mutant male; BLK = blank.

**Caution:** Ensure that all reagents are at room temperature for at least 30 min before conducting the assay.

2. Catalyst addition: Add 50 μL of catalyst to each well containing the sample or standard. Ensure thorough mixing by gentle pipetting.

3. Incubation: Incubate the plate for 5 min at room temperature to allow the reaction between the sample and catalyst.

4. DPS solution addition: After incubation, add 100 μL of DPS solution to each well. Cover the plate's reaction wells with aluminum foil to protect them from light.

5. Incubation for fluorescence development: Incubate the plate at room temperature for 45 min to allow fluorescence to develop in response to the reaction.

6. Fluorescence measurement: Using the Agilent BioTek Synergy H1 plate reader, excite the samples with 480 nm wavelength and measure emissions at 530 nm wavelength.

**Critical:** It is recommended to conduct a preliminary trial run using several sample dilutions prior to performing the actual assay to determine the appropriate sample concentration causing fluorescence readings to fall within the range of H_2_O_2_ standards. Repeat the test using samples made from a greater number of flies if the samples are too diluted. If the samples are highly concentrated, dilute them as necessary.

**C. Protein quantification using BSA**

1. Standard solution preparation: Prepare standard BSA solutions with concentrations appropriate for the assay. Dispense 2 × 5 μL of each standard solution per well in a clear-bottom 96-well plate. See Table 5 for an example of a plate layout for protein quantification.

**Table 5.** Plate setup: Layout of standards, samples, and blank for protein quantification

	1	2	3	4	5	6	7	8	9	10	11	12
A	ST.1	ST.1	CF1	CF1	CM1	CM1						
B	ST.2	ST.2	CF2	CF2	CM2	CM2						
C	ST.3	ST.3	CF3	CF3	CM3	CM3						
D	ST.4	ST.4	MF1	MF1	MM1	MM1						
E	ST.5	ST.5	MF2	MF2	MM2	MM2						
F	BLK	BLK	MF3	MF3	MM3	MM3						
G												
H												

ST. = standard; CF = control female; MF = mutant female; CM = control male; MM = mutant male; BLK = blank.

2. Sample addition: For each biological replicate, add 5 μL of the sample into separate wells of the 96-well transparent plate. Ensure two technical replicates for each sample.

3. Bradford reagent addition: Add 200 μL of Bradford reagent (at room temperature) to each well containing the standard solution or sample. Mix the contents thoroughly by pipetting five times. Seal the plate with parafilm to prevent evaporation during incubation.

4. Incubation: Incubate the plate for 30 min at room temperature to allow the Bradford reagent to react with proteins in the standard solutions and samples.

5. Absorbance measurement: After incubation, measure the absorbance of the samples at 595 nm wavelength using the Agilent BioTek Synergy H1 plate reader. Record the absorbance readings for each well to quantify protein concentrations in the samples based on the standard curves generated.

**D. Measuring fluorescence and absorbance using Agilent BioTek Synergy H1**

1. Ensure all the samples are properly placed and mixed in a 96-black well plate with the appropriate concentration.

2. Power on the BioTek Synergy H1 plate reader and the connected computer.

3. Open the Gen5 Data Analysis Software.

4. Ensure that the instrument completes its initialization and calibration routines.

5. Open Gen5 3.14 software. Select *New* and then *Read*.

6. Define the plate layout (e.g., sample wells, control wells, blank wells).

7. Choose *Fluorescence* as the detection mode. Enter the excitation and emission wavelengths at 480 and 530 nm, respectively, for fluorescence measurement. Choose *Absorbance* as the detection mode for protein quantification and enter 595 nm as the wavelength.

8. Place the microplate in the instrument's plate holder.

9. Click the *Read* button in the software to begin fluorescence or absorbance measurement. You can monitor the progress on the software interface.

10. Once the run is complete, retrieve the fluorescence intensity or absorbance data from the software. Use the Gen5 software or export the data to Excel for further analysis.

*Note: Samples with signals exceeding the highest standard should be diluted in PBS and reanalyzed, and the concentration should be adjusted by the dilution factor. Average duplicate readings for each standard, control, and sample. Subtract the mean blank value from all readings to calculate corrected fluorescence or absorbance.*

## Data analysis

**Further analysis**

1. The corrected fluorescence or absorbance values for each standard are plotted against the final concentration of H_2_O_2 _or BSA, respectively.

2. The trendline is obtained through these points to create the standard curve.

3. The trendline equation is calculated providing the most accurate fit based on the obtained standard curve data.

4. The corrected sample fluorescence or absorbance reading is applied to the standard curve to determine the H_2_O_2_ or protein concentration in the sample wells.

5. For fluorescence measurement, the normalized concentration of H_2_O_2_ in the test samples is calculated using the formula:

Normalized H_2_O_2_ concentration (μM/μg protein) = (C × D)/P

where:

*C* is the H_2_O_2_ concentration (μM) derived from the standard curve.

*P* is the protein content in the sample well (μg).

*D* is the dilution factor applied to the sample before analysis.

This calculation expresses the H_2_O_2_ concentration relative to the protein content of the sample. The total free radicals in the samples are expressed as μM H_2_O_2_/μg protein.

**Statistical analysis using GraphPad Prism**

1. Select the analysis type: In GraphPad Prism or other graphing software, use an unpaired t-test, paired t-test (if measurements are related), or other appropriate statistical tests based on the experimental design.

2. Input data: Input the ROS level data for the control and mutant groups of specific sexes into the designated sections in GraphPad Prism. Specify the grouping variable (control vs. mutant) and ensure the data are correctly assigned to their respective groups.

3. Perform the analysis: Run the statistical analysis using the selected test (e.g., unpaired t-test).

4. Review results: Examine the results generated by GraphPad Prism, including the t-value, degrees of freedom, p-value, and confidence intervals.

5. Create graphs: Statistical analysis of free radical levels in various samples can be performed using the GraphPad Prism software. Typically, a t-test, ANOVA, or a modified version of both are appropriate tests. We presented the results of a t-test based on our experimental data. Using GraphPad Prism, we created a bar graph with standard error of the mean (SEM) as error bars and included it as an example in the text. It is up to the author to provide information in the graph, such as the number of flies used per sample (n), p-value, * symbols to indicate statistical significance, etc.

6. Include statistical details: Ensure that the graphs include statistical details, such as significance asterisks or letters indicating levels of significance (e.g., *** or a, b, c) based on the results of the t-test.

## Validation of protocol

**Figure 2.**
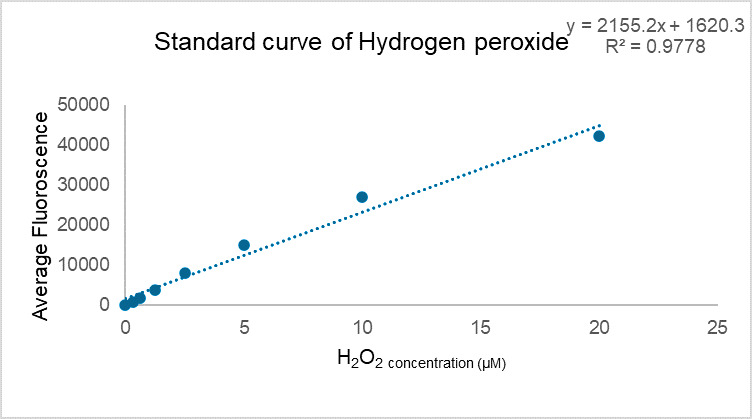
Standard curve of hydrogen peroxide

**Figure 3.**
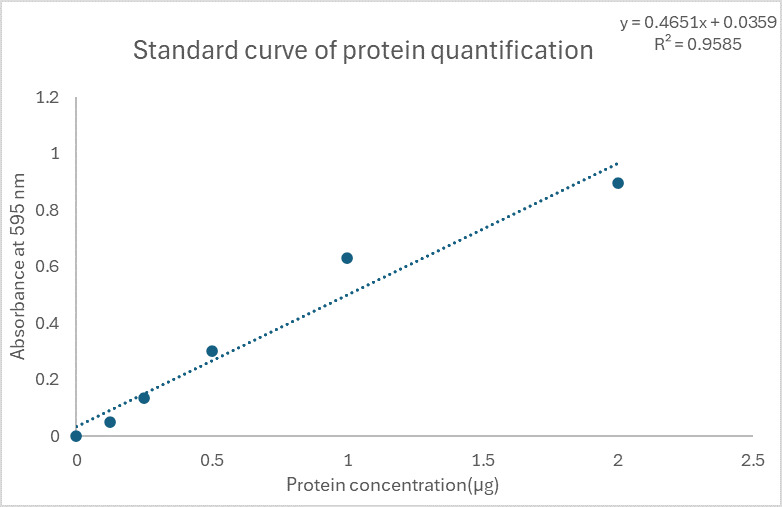
Standard curve for protein quantification

Figure 2 demonstrates typical results of assays quantifying ROS/RNS free radicals and Figure 3 demonstrates the result of the assay for protein quantification. Fluorescence measurement was performed on the Agilent BioTek synergy H1 plate reader. Use a 480/530 nm filter set with a 530 nm cutoff. The data provided is solely for reference and should not be used to interpret actual results.

## General notes and troubleshooting

**General notes**

1. All solutions, including DCFH and protein quantification reagents, should be freshly prepared on the day of the experiment to ensure accuracy and reproducibility.

2. Maintain all solutions at low temperatures during the process.

3. The accuracy of ROS quantification depends on minimizing background fluorescence and ensuring uniform sample distribution in the 96-well plate. Using black-walled plates and proper pipetting techniques can help reduce variability.

**Troubleshooting**

**Problem 1:**

Inconsistent mixing of reagents, improper plate handling, or uneven distribution of samples and reagents can lead to variability in fluorescence readings across the wells of the plate.

**Potential solution:**

• Thoroughly mix all reagents after adding them to the wells by pipetting a few times to ensure uniformity.

• Seal the plate to prevent evaporation and contamination during incubation and measurement.
